# Beyond the Usual: Pantoprazole-Induced Anaphylaxis During Peribulbar Anesthesia

**DOI:** 10.7759/cureus.38738

**Published:** 2023-05-08

**Authors:** Antonio Palha Ribeiro, Ana Gonçalves, Carolina Mateus, Carlos Mexêdo

**Affiliations:** 1 Anesthesiology and Critical Care, Centro Hospitalar Universitário de Santo António, Porto, PRT; 2 Anesthesiology and Critical Care, Centro Hospitalar Universitario de Santo António, Porto, PRT

**Keywords:** adrenaline, peribulbar anesthesia, proton-pump inhibitor, pantoprazole, anaphylaxis

## Abstract

Proton-pump inhibitors (PPIs) are one of the most prescribed drugs in the world. Although they are remarkably safe, with minimal adverse effects, it has rarely been reported as a cause of anaphylaxis. Hence, we report the case of a 69-year-old patient who experienced intravenous pantoprazole-induced anaphylaxis during peribulbar block anesthesia for mechanical vitrectomy.

## Introduction

Anaphylaxis is an uncommon incident and is defined as a severe and potentially life-threatening generalized or systemic hypersensitivity reaction that can occur in multiple environments including during anesthesia [1]. It is estimated that perioperative anaphylaxis occurs in approximately one in 10,000 anesthetics mostly caused by antibiotics, particularly penicillins, neuromuscular blocking agents, notably rocuronium and reversal agents such as sugammadex [2].

Proton-pump inhibitors (PPIs) are one of the most prescribed drugs in the world and are recommended preoperatively in patients at risk of pulmonary aspiration to prevent stress ulcers [3]. They are remarkably safe, with minimal adverse effects, most of which are related to the drug’s pharmacokinetic interaction profiles [4]. Nevertheless, hypersensitivity reactions can still occur, although it has rarely been reported as a cause of anaphylaxis. Hence, we report the case of a patient who experienced intravenous pantoprazole-induced anaphylaxis.

## Case presentation

A 69-year-old woman, American Society of Anesthesiologists (ASA) Physical Status II, was presented with a history of arterial hypertension, dyslipidemia, obesity class I and a surgical history of bilateral knee arthroplasty. The patient denied any history of known medication allergies. She was scheduled for outpatient mechanical vitrectomy due to retinal detachment. The preoperative assessment of the patient was done, and it was documented that her height was 157 cm, and she weighed 81 kg with a BMI of 31.3 kg/m^2^. Hemodynamically, the patient was stable with tensional values of 109/60 mmHg and other vital signs in the normal range: heart rate, 65 bpm; oxygen saturation, 96% at room air; and tympanic temperature, 36.6ºC. Standard ASA monitoring was performed, and an 18G IV catheter was inserted.

Peribulbar block was performed with 7 ml of ropivacaine 1% (5 ml inferotemporal injection and 2 ml superonasal injection) without any reported complications related to the technique. At the end of the procedure (around 75 minutes), 40 mg of intravenous (IV) pantoprazole and 200 mg of IV hydrocortisone as well as subconjunctival cephazolin were concomitantly administered. Approximately two minutes after administration of these drugs, the patient developed transient loss of consciousness with desaturation (peripheral capillary oxygen saturation (SpO_2_) ~85%); heart rate of 110bpm; blood pressure of 80/45 mmHg; and an exuberant facial, cervical and thoracic rash.

A systematic ABCDE evaluation of the patient revealed a patent airway, SpO_2_ 90% with high concentration oxygen mask and pulmonary auscultation with symmetric vesicular murmur bilaterally with scattered wheezing. Additionally, a progressive hypotensive profile was noted with poor peripheral perfusion and prolonged capillary refill time with minimal arterial pressure of 40/20 mmHg and heart rate of 121 bpm with a Glasgow Coma Scale of 4 (O2V1M1), without other relevant changes. It was assumed an anaphylactic shock and was promptly administered 0.5 mg of intramuscular (IM) adrenaline, 2 mg of IV clemastine, 200 mg of IV hydrocortisone and a 500 ml fluid bolus of balanced crystalloid solution was started with gradual improvement of arterial pressure and oxygen peripheral saturation (SpO_2_ > 95%). A blood gas analysis was then performed (Table 1).

**Table 1:** Blood gas analysis with patient's values and its reference range pO_2_: partial pressure of oxygen; pCO_2_: partial pressure of oxygen of carbon dioxide; SpO_2_: peripheral capillary oxygen saturation; HCO_3-_: bicarbonate.

Pertinent Lab	Value	Reference Range
pH	7.369	7.35-7.45
pO_2_	116 mmHg	75-100 mmHg
pCO_2_	34.8 mmHg	32-45 mmHg
SpO_2_	92.5%	92%-98.5%
HCO_3_-	20 mmol/l	22-26 mmol/l
Anion gap	11.2 mmol/l	8-12 mmol/l
Lactate	0.7 mmol/l	0.5-2 mmol/l
Na	139 mmol/l	135-148 mmol/l
K	3.7 mmol/l	3.5-5.3 mmol/l
Ca	1.13 mmol/l	1.13-1.32 mmol/l

The patient was then transferred to the postanesthesia care unit and remained hemodynamically unstable with an arterial blood pressure of 50-60/20-30 mmHg so a second bolus of 0.5 IM adrenaline was administered. A second large bore 16G IV catheter was placed in the dorsum of the left hand followed by the placement of a 20G right radial arterial catheter. Despite an initial response, a progressive decline of arterial blood pressure (minimum systolic of 50 mmHg) refractory to fluidotherapy (total of 2 liters of crystalloids) occurred, so it was decided to start vasopressor support with IV noradrenaline (maximum dosage of 1.2 mg/h). After a few minutes, an improvement in arterial pressure was seen. Simultaneously, serum tryptase measurement was collected after 45 minutes of the onset of symptoms that was found to be increased at 54.5 µg/L (normal value < 11.4 µg/L). The patient was also observed by ophthalmology and the team elected to remove the eye ointment placed during the procedure and performed an eye wash, although no relevant changes were identified.

The patient was then transferred to the intensive care unit for monitorization, with good clinical evolution under IV corticotherapy and clemastine with complete resolution of organ dysfunctions. After 24 hours of the episode, IgE and serum tryptase values were within normal range. Allergy detection IgE blood tests for penicillin G, penicillin V, ampicillin, amoxicillin and cefaclor were also all negative. She was discharged 72 hours after admission medicated with 10 mg of oral prednisolone and referred to allergology. The working diagnosis was assumed to be an anaphylactic shock most probably caused by cephazolin, despite negative IgE blood tests. The patient was observed by allergology three months later; they recommended cessation of beta-lactams with future indication to perform cutaneous tests for beta-lactams.

Two months later after an allergology consultation in which cutaneous tests were negative for all beta-lactams including cephazolin, the patient was admitted to the emergency department with diffuse abdominal pain associated with multiple episodes of diarrhea and vomiting, compatible with acute gastroenteritis. It was decided to administer 1,000 mg of IV acetaminophen for pain control and 40 mg of IV pantoprazole, and a few minutes after, the patient quickly developed a maculopapular rash located in the upper limbs and thorax with complaints of generalized pruritus. The patient did not show any signs of respiratory distress, with SpO_2_ of 91% room air and no changes in pulmonary auscultation remaining hemodynamically stable with a heart rate of 117 bpm. It was assumed an allergic reaction to pantoprazole and administered 200 mg of IV hydrocortisone and 2 mg of IV clemastine. She was discharged from the hospital six hours later with the total resolution of the symptoms. 

**Figure 1:**
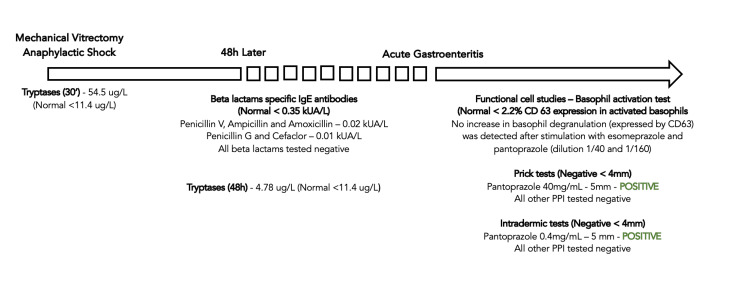
Immunological and allergy tests performed for identification of anaphylaxis culprit agent

Three months later, the patient was observed in the allergology consultation and it was decided to perform a basophil degranulation test (evaluated by CD63 expression) after esomeprazole and pantoprazole stimulation was negative. Consequently, prick-prick and intradermic tests were done for all the proton-pump inhibitors (omeprazole, esomeprazole, pantoprazole, rabeprazole and lansoprazole), which were positive for pantoprazole (40 mg/ml, dilution 1/1 in prick-prick test, and 0.4mg/ml, dilution of 1/100 in epicutaneous test) and negative for all the other PPI. It was recommended to total eviction of all PPI and to resort to alternative groups such as antihistamines and antacids if clinically indicated. Immunological and allergy tests performed for the identification of the anaphylaxis culprit agent are shown in Figure 1.

## Discussion

Anaphylaxis is a life-threatening emergency that requires early recognition of signs and symptoms, and immediate treatment should be directed. In this case, a prompt diagnosis of anaphylaxis was made, as the patient showed exuberant skin signs that co-existed with respiratory, cardiovascular and neurologic dysfunctions, administering immediately intramuscular adrenaline and aggressive volume replacement. Additionally, serum tryptase measurement was collected as followed by the European Academy of Allergy and Clinical Immunology (EAACI) anaphylaxis guidelines in the first and 36th hour after the onset of symptoms [5]. As an acute elevation of serum tryptase in the first hour reverted to the normal range in the 36th hour was observed, the diagnosis of anaphylaxis was confirmed because high tryptase levels indicate degranulation of mast cells, which, in this case, most probably occurred by an IgE-mediated mechanism due to the immediate reaction observed [6].

Moreover, the first essential step in the prevention of anaphylaxis is the identification of the causative agent. In this case, there were only four possible agents that could have caused anaphylaxis. Ropivacaine is an amide local anesthetic widely used for locoregional anesthesia that can rarely cause anaphylactic reactions. However, in this case, the onset of symptoms was around 75 minutes after administration of this anesthetic which makes it unlikely as the contributing agent. Hydrocortisone was also administered at the end of the surgery simultaneously with subconjunctival cephazolin and pantoprazole and could have also been the trigger of the anaphylaxis. Nevertheless, hydrocortisone was readministered for treatment management, and no recurrent anaphylaxis was observed.

Pantoprazole could have also been the culprit of the anaphylaxis, although it was mistakenly considered highly unlikely due to the relative iniquity of this drug. Cephazolin is the most common cause of perioperative anaphylaxis, and as this patient had a recent history of knee arthroplasty where she received cephazolin without any reported allergy, it was assumed that she could have been sensitized in that period. As a consequence, she could have developed a severe allergy to this antibiotic in the vitrectomy, even if the route of administration was subconjunctival, as there has been at least one report of cephazolin-induced anaphylaxis through this route [7].

Moreover, it was presumed that cephazolin would have been the causative agent of the anaphylactic shock even though IgE blood measurements of multiple beta-lactams were all negative, strongly biased by the fact that antibiotics are the most common drugs involved in perioperative anaphylactic reactions. Therefore, no further investigation was performed on the other possible drugs, incorrectly assuming that the patient had an allergy to cephalosporins. An allergic reaction after the patient was admitted to the emergency department for acute gastroenteritis months later was the turning point as the only common drug administered in both episodes was pantoprazole; so a detailed investigation was performed in order to confirm if this proton-pump inhibitor was responsible for these incidents.

There are multiple tests to corroborate the allergy suspicion which include skin tests (prick tests and intradermic tests) and oral provocation tests. More recently, basophil activation tests by CD63 expression have been reported to be a promising complementary tool for inclusion in the allergological work-up as it is not invasive and has an excellent specificity [8]. In this case, it was decided to perform this test in the first place but due to its moderate sensibility, skin tests were necessary, confirming a pattern of three cross-reactivity as previously described by Benito-Garcia et al., which means the patient had a hypersensitivity reaction to a single PPI alone (pantoprazole), with tolerance of other PPIs [9]. This complete investigation was of extreme importance because this enables the patient to take safely other PPIs in the future, considered to be more efficient than other class of drugs such as antihistamines and antacids in specific situations such as *Helicobacter pylori* eradication and chronic gastritis. A few case reports of pantoprazole-induced anaphylaxis have been published in the literature; most of them were caused by oral administration and also three cases of intravenous administration [10-12], the latter representing the only case during general anesthesia. As far as we know, this is the first case report of pantoprazole anaphylaxis during locoregional anesthesia.

## Conclusions

PPIs' anaphylaxis incidence is expected to increase in the next years not only because of the crescent awareness of clinicians but also due to the rising consumption of this class of drugs. Nevertheless, as anaphylaxis is still a rare adverse effect of these drugs, it is necessary to maintain a high level of suspicion especially when this drug is administered simultaneously with other drugs, which happened in this case delaying the confirmation of the causative agent. This case not only highlights the importance of anesthesiologists in the prompt management of emergent incidents such as anaphylaxis but also takes into consideration that cognitive bias in our daily practice can result in incomplete investigations that may lead to avoidable life-threatening consequences for our patients.

## References

[1] Johansson SG, Hourihane JO, Bousquet J (2001). A revised nomenclature for allergy: an EAACI position statement from the EAACI nomenclature task force. Allergy.

[2] Harper NJ, Cook TM, Garcez T (2018). Anaesthesia, surgery, and life-threatening allergic reactions: epidemiology and clinical features of perioperative anaphylaxis in the 6th National Audit Project (NAP6). Br J Anaesth.

[3] (2017). Practice guidelines for preoperative fasting and the use of pharmacologic agents to reduce the risk of pulmonary aspiration: application to healthy patients undergoing elective procedures: an updated report by the American Society of Anesthesiologists Task Force on preoperative fasting and the use of pharmacologic agents to reduce the risk of pulmonary aspiration. Anesthesiology.

[4] Esplugues JV, Marti-Cabrera M, Ponce J (2006). Safety of proton pump inhibitors. (Article in Spanish). Med Clin (Barc).

[5] Muraro A, Worm M, Alviani C (2022). EAACI guidelines: anaphylaxis (2021 update). Allergy.

[6] (2011). Anaphylaxis: assessment to confirm an anaphylactic episode and the decision to refer after emergency treatment for a suspected anaphylactic episode: NICE clinical guideline 134. http://www.nice.org.uk/guidance/cg134.

[7] Berrocal AM, Schuman JS (2001). Subconjunctival cephalosporin anaphylaxis. Ophthalmic Surg Lasers.

[8] Laguna JJ, Bogas G, Salas M (2018). The Basophil Activation Test can be of value for diagnosing immediate allergic reactions to omeprazole. J Allergy Clin Immunol Pract.

[9] Benito-Garcia F, Chambel M, Morais-Almeida M (2018). Anaphylaxis due to proton pump inhibitors: current understanding and important clinical considerations. Expert Rev Clin Immunol.

[10] Faridaalaee G, Ahmadian Heris J (2018). Anaphylaxis as a rare side effect of pantoprazole: a case report. Emerg (Tehran).

[11] Yousefi H, Moayedi S, Harorani M, Sahebi A, Golitaleb M (2019). Anaphylaxis as a side effect of pantoprazole. Shiraz E-Med J.

[12] Lai HC, Hsu SW, Lu CH, Ma HI, Cherng CH, Hung NK, Wu CT (2011). Anaphylaxis to pantoprazole during general anesthesia. J Anesth.

